# Pragmatic clinical trials in the context of regulation of medicines

**DOI:** 10.1080/03009734.2018.1515280

**Published:** 2018-09-25

**Authors:** Rolf Gedeborg, Charles Cline, Björn Zethelius, Tomas Salmonson

**Affiliations:** Swedish Medical Products Agency, Uppsala, Sweden

**Keywords:** Drug approval, government regulation, methods, pharmaceutical preparations, pragmatic clinical trial

## Abstract

The pragmatic clinical trial addresses scientific questions in a setting close to routine clinical practice and sometimes using routinely collected data. From a regulatory perspective, when evaluating a new medicine before approving marketing authorization, there will never be enough patients studied in all subgroups that may potentially be at higher risk for adverse outcomes, or sufficient patients to detect rare adverse events, or sufficient follow-up time to detect late adverse events that require long exposure times to develop. It may therefore be relevant that post-marketing trials sometimes have more pragmatic characteristics, if there is a need for further efficacy and safety information. A pragmatic study design may reflect a situation close to clinical practice, but may also have greater potential methodological concerns, e.g. regarding the validity and completeness of data when using routinely collected information from registries and health records, the handling of intercurrent events, and misclassification of outcomes. In a regulatory evaluation it is important to be able to isolate the effect of a specific product or substance, and to have a defined population that the results can be referred to. A study feature such as having a wide and permissive inclusion of patients might therefore actually hamper the utility of the results for regulatory purposes. Randomization in a registry-based setting addresses confounding that could otherwise complicate a corresponding non-interventional design, but not any other methodological issues. Attention to methodological basics can help generate reliable study results, and is more important than labelling studies as ‘pragmatic’.

## What is a pragmatic study?

A distinction is sometimes made between explanatory and pragmatic clinical trials. While explanatory trials aim to estimate the efficacy of an intervention under optimized conditions, the term pragmatic trials usually refers to what an intervention accomplishes when applied in wider clinical practice (1). The traditional phase III randomized clinical trial in a drug development programme would typically be an explanatory trial, focusing on establishing an ideal situation to estimate the efficacy of the drug in relation to a sufficiently well characterized safety profile. The pragmatic clinical trial concept addresses questions with a focus on estimating effects in a setting closer to routine clinical practice and sometimes using routinely collected data. The pragmatic trial is therefore expected to more adequately inform a clinical or policy decision by providing evidence more relevant for use of a medicine in daily clinical practice. This is sometimes also referred to as ‘real-world evidence’ (2). An evaluation tool (PRECIS-2) has been proposed to allow a structured grading of specific domains that defines explanatory versus pragmatic aspects of a trial (3). It tries to quantify aspects such as how similar the participants in the trial are to the target population in routine clinical practice, and how much extra effort is made to recruit patients compared to the usual care setting. The differences in setting where the trial is conducted, differences in the resources needed to deliver the intervention, and measures applied to promote adherence to treatment are also compared to expectations from routine clinical care. Questions on how closely participants are followed up, how relevant the outcome measure is to participants, and completeness of data are also assessed in the PRECIS-2 tool. A pragmatic trial might therefore use less strict control over which patients to include, the exposure, and standard of care. Study procedures may not actively promote adherence to treatment, and may accept that follow-up of patients and measurement of outcomes are not fully standardized and optimal (4). The design and conduct of pragmatic trials offer distinct challenges, and the appropriate framework for this type of studies is under development (5).

For the purpose of the present discussion we will not consider non-interventional studies or single-arm studies that may also be considered pragmatic designs, but instead focus on randomized trials. Some aspects of pragmatic trials are of particular relevance or concern from a regulatory perspective. Protection of human subjects is of key importance, e.g. concerning informed consent and compliance with good clinical practice (GCP) (6). The concept of ‘low-intervention’ clinical trials has therefore been introduced in the legislation, to allow a risk-based approach to using less rigid rules, e.g. as regards monitoring and traceability of investigational medicinal products (7). These aspects are of major importance but not further discussed in this article.

It is apparent that there is no distinct boundary between explanatory and pragmatic trials (4). Both types of study can answer relevant questions, and the most appropriate study design should therefore be determined based on the specific scientific question to be answered.

## Are pragmatic studies of value for regulators?

One important requirement for the approval to market a medicinal product is having sufficient evidence that the expected beneficial effects outweigh potential risks in a defined population. This conclusion on the benefit–risk balance is always expected to be reconsidered whenever new relevant evidence becomes available during the life-cycle of a medicinal product (8). Even if there are large studies underlying a marketing authorization, there will never be enough patients studied in all subgroups that may potentially be at higher risk for adverse outcomes, or sufficient patients to detect rare adverse events, or sufficient follow-up time to detect late adverse events that require long exposure times to develop. There is therefore always a need for continued safety surveillance and often also a need for systematic post-marketing studies of specific safety concerns.

Sometimes there is also uncertainty regarding the efficacy of the new drug that warrants further studies post approval. It may not be feasible, or even necessary, to require large randomized clinical trials before making a decision to approve a new drug or a new indication. The degree of certainty in the characterization of effects that is considered necessary to obtain before approval must be weighed against the urgency of the unfilled medical need for the product, in order to make new treatments available to patients as soon as possible. Some drugs are therefore approved without having large randomized trials documenting efficacy and safety. Over the period 1 January 1999 to 8 May 2014 the European Medicines Agency (EMA) approved 44 new indications for 35 drugs without a randomized controlled study, among 415 approvals of new indications reviewed (9). Generic drugs, biosimilars, diagnostics, medical devices, vaccines, antimicrobials, blood products, and fixed-dose combinations of existing products were excluded from this review. The majority of the new indications approved without a randomized controlled study were for haematological malignancies, oncology, and metabolic conditions. Such approvals may be possible if the natural course of disease allows for the isolation of drug effects without a comparator. Though the effect size in the target population may remain uncertain due to the lack of reliable calibration against a standard of care, such limitations may be acceptable in often fatal orphan diseases, for which there are no other therapeutic options, or situations when an ethically acceptable control group cannot be specified. Specific legal frameworks have been created for conditional approval and approval under exceptional circumstances, to be used in situations when data are limited but medical need is great. Obligations to perform further clinical studies post approval are then imposed on the drug company. These studies may e.g. focus on endpoints different from the primary endpoints of the studies available at the time of initial authorization, evaluate longer treatment duration, and/or the number of subjects receiving the product as part of imposed studies may be higher compared to studies available before approval (10). In some cases data in closely related patient populations also contribute to the generation of comprehensive data in support of the granted indication, and it is not uncommon that the data from post-marketing trials after conditional approval have led to a change in the definition of target population in the therapeutic indication (10). It may therefore be relevant that post-marketing trials have more pragmatic characteristics, if this is needed to provide the information on efficacy and safety that is missing. From a regulatory perspective, both explanatory and pragmatic trials are therefore relevant.

## Potential concerns with pragmatic trials from a regulatory perspective—the completeness and validity of the data from registries and health records

One key feature of a pragmatic trial may be that data collection is based largely on routinely available data in electronic health records or disease registries (11). There are some distinct advantages in terms of reduced effort for both investigators and study subjects, and consequently reduced cost. This may facilitate recruitment of patients to the trial and enable a larger sample size. The potential concerns lie in the validity and completeness of data.

A pragmatic non-interventional study, e.g. conducted using prescription and/or disease registries, may seem appropriate in that it is representative of use in clinical practice. A prescription registry, however, often does not include medications administered in-hospital. This may be a major limitation since many drug treatments are initiated in-hospital, and hospitalized patients may be particularly frail and prone to adverse events. Another example is the use of hospital discharge registries where primary care encounters are not registered (12), or registers based on general practitioner data, where data from patients cared for in hospital-based specialist clinics are not available unless linked with hospital data (13,14). The potential problem that the definition of the study population can introduce selection bias therefore needs to be considered irrespective of the type of study, also for non-interventional studies. Selection bias can present major limitations also for studies labelled as pragmatic with a very permissive inclusion of patients.

The completeness of data collection is a key issue for any type of trial and in focus for GCP (6). An example is when data on outcomes routinely collected in the electronic patient records are not accessible during the study and are found to be frequently missing during analyses (15). A study design and study conduct that prevents missing information is always preferable to statistical handling of missing information at the analysis stage (16). Such methods often have unverifiable assumptions. Sensitivity analyses to evaluate the impact of the handling of missing data and associated assumptions are essential for the interpretation of study results. The importance of choosing an appropriate estimand during the planning stage of a study, so that attempts to prevent missing data can be tailored to that choice, and appropriate estimation methods can be specified, must be stressed (17). It is essential to detect and adequately handle intercurrent events, such as use of alternative treatment, discontinuation of treatment, and treatment switches, in the analyses. Otherwise, these types of events may lead to invalid conclusions regarding treatment effects (17). These two fundamental considerations in the study design, how to prevent missing information and defining the appropriate estimand, may be a greater concern for pragmatic study designs.

Another risk with the use of routinely collected health-care data is that the measurement of outcomes may be a less precise. For an outcome such as long-term all-cause mortality this is usually not a major problem. Mortality is a well-defined outcome that can be reliably captured by administrative data. For outcomes with insidious onset, or outcomes requiring accurate measurements and strict definitions, this can on the other hand be a substantial problem. An example is the difficulty to identify severe infections based on hospital discharge diagnoses in health-care databases (18). Patients with a well-defined diagnosis such as meningitis, often also being the primary reason for hospitalization, can be accurately identified using such a data source. This is in contrast to a common and important diagnosis such as sepsis, which is a less well-defined condition often complicating a hospital stay rather than being the primary cause for admission. The sensitivity with which patients with sepsis can be identified from hospital discharge diagnoses is expected to be very low (18).

The use of randomization integrated in a registry-based setting is an important step towards increased reliability of patient-relevant research aimed to reflect clinical practice (19). Randomization offers the unique property of addressing not only known and measured confounding, but also taking care of unmeasured and unknown confounding. It should, however, be recognized that randomization does not address any other methodological problem discussed in this article, such as selection bias or misclassification. Adding randomization in a registry-based setting should consequently be seen as a means to address confounding that could complicate the corresponding non-interventional study design. Other methodological issues remain, however.

## To what extent does the study population in randomized clinical trials need to be representative of the target population?

An argument in favour of a pragmatic approach for clinical studies is that this can make the results generalizable to a wider target population, i.e. the population where the results of the study are expected to be applicable. It is, however, not an imperative feature for a comparative study that the study population accurately mirrors the target population in terms of the distribution of relevant patient characteristics (20). This thinking most likely stems from the process of survey-sampling. In a purely descriptive study, such as a survey, it is essential to have a representative sample of the population of interest. In an inferential comparative study, this may instead hamper the ability to make internally valid inferences (20). Concerns regarding generalizability are only meaningful once the results are deemed internally valid, i.e. that they are sufficiently unbiased to warrant causal inference, for the restricted study population.

In a comparative study the assumption of homogeneity of the effect in the study population is central to a data analysis that ultimately presents an overall effect estimate for the study population. In this situation the result is assumed to be the same in all parts of the distributions of all relevant patient characteristics that are well represented in the study population. This means that e.g. the age distribution need not be exactly the same as in the target population but all parts of the age distribution in the target population must be reasonably well represented in the study population. This could justify over-sampling, e.g. of some parts of the age distribution, in order to have sufficient representation of all parts of the relevant age range for the target population in the source population for the study (20). It is also important to remember that this assumes that there is no effect modification, meaning that the conclusions of therapeutic efficacy (and safety) apply consistently across relevant subgroups of the clinical trial population.

An obvious example is that all relevant age categories must be adequately represented in the study population. If one states that results from an explanatory randomized trial are unreliable regarding their prediction of expected effect in the overall target population, this is the same as stating that there is important effect modification by some patient characteristic. Consequently, further efforts should aim to characterize this effect modification, and not simply estimate another overall effect estimate. If clinical data fail to establish statistically persuasive evidence of effect, there may be an interest in exploratory analyses to further characterize the effect in relevant subgroups (21). It is otherwise difficult to understand how to interpret the totality of evidence. From a regulatory perspective there is a focus on identifying the patient population where the benefits outweigh the risks. The problem with exploring subgroups is closely related to the problem of multiple testing, and carries an increased probability of false-positive findings. It is therefore important to conduct reliable analyses of potential effect modification from e.g. age, renal, or hepatic function, whether through direct such analyses of interaction or through pre-planned subgroup analyses (21).

## How can the results be generalized outside the study population in an explanatory phase III trial?

A study aiming for high internal validity through an explanatory type of trial design may for this purpose select a homogeneous population that can differ in important ways from the intended broad target population in clinical practice. These studies have therefore sometimes been questioned for uncertainty about generalizability of the results.

Extrapolation of the study results is always needed to some extent, otherwise they are not meaningful. There is a need to generalize the results at least in time, so that we consider them valid not only for the patients studied but also for future patients. Although this may be seen as self-evident, there may be an evolution of the treatment effect over time (22). This is something that may need to be considered in cases where data have evolved over a period of time in parallel to changes in the population and in health care, i.e. new diagnostic criteria and treatment guidelines, and in an evaluation that should be based on the totality of data.

Apart from generalizability over time there is often a need for extrapolation also in relation to other characteristics of the target population. A common example is age, and to what extent results can be generalized to the extremes of age—children and the elderly.

The basis for extrapolation to a paediatric target population rests on the relevance of results obtained in an adult study population. This can be quantified in terms of drug exposure being dependent on body size and organ maturation (pharmacokinetic data), differences in pharmacodynamic response, and age-dependent differences in disease characteristics (23).

Simply generating an overall effect estimate including all age groups, children as well as adults, is clearly not appropriate. Extrapolation is in some instances not justifiable, while in other cases extrapolation is uncomplicated, based on well-established understanding of the drug pharmacology and the disease (23). It becomes evident that a targeted approach to data collection is needed, so that children participate only in trials with specific objectives to obtain the information needed for a specific intervention and patient group. The aim should be to characterize patient factors that influence the efficacy and safety of the drug, and not only to estimate an overall effect size.

A relevant aim for a post-marketing trial could also be to further elucidate the effects in an elderly population, if they were not adequately represented in the populations already studied. If this is the concern, then it may be more effective to target a study specifically at this subpopulation, rather than performing a wide pragmatic clinical trial covering the entire target population and all age groups (24). In this scenario an overall effect estimate for the entire target population may be difficult to interpret. To clarify if the effect is age-dependent the study must be dimensioned specifically with the question of potential effect modification by the age in focus (21). The overall effect estimate cannot be the only aim of the study. It becomes more important to understand the impact of patient characteristics, such as age, body size, and renal and hepatic function, rather than to calculate an overall effect estimate in a broad population representative of the entire target population.

## Pragmatism may make a randomized trial more relevant for questions relating to using the medicine in clinical practice and also less expensive, but comes at a cost from other aspects

An example of how pragmatic trials can be questioned based on their pragmatic design is provided by the regulatory procedures concerning potential adverse effects of hydroxyethyl starch (HES) products finalized in 2013 (25). Key evidence in these procedures were some academic clinical trials that could be considered pragmatic (26,27). Criticism against these studies focused on difficulties with defining and controlling the exposure in these studies, and the study population being poorly defined due to the broader pragmatic inclusion of patients (28). In a regulatory evaluation it is important to be able to isolate the effect of the product or substance in focus of the investigation, and to have a defined population that the results can be referred to. A feature such as having a wide and permissive inclusion of patients might therefore actually hamper the utility of the results for regulatory purposes.

Simplifying the data collection process in a clinical study is expected to require trade-offs, but the actual consequences must be evaluated for each specific outcome in relation to the research question at hand. Is the accuracy of data sufficient to support reliable conclusions from the study? It may e.g. be problematic to claim effectiveness of an intervention without reliable concomitant estimation of relevant safety endpoints. Safety reporting in published randomized trials has been found to be variable but largely inadequate (29). This is an area where simplification of data collection may be a concern, particularly for new medicines. For a study investigating a well-characterized medicine, e.g. where the investigational medicinal product is covered by a marketing authorization, and the quality, safety, and efficacy have already been assessed in the course of the marketing authorization procedure, the intervention poses only very limited additional risk to the subject compared to normal clinical practice. Such ‘low-intervention’ clinical trials may be of crucial importance for assessing standard treatments and diagnoses, thereby optimizing the use of medicinal products. Those clinical trials could be subject to modified requirements, e.g. as regards monitoring and traceability of investigational medicinal products (19). In other situations reliable collection of all relevant data according to GCP remains essential.

## Conclusion

Regulatory decision-making should always be based on the totality of data and not on whether a specific study is labelled as explanatory or pragmatic. The regulators have to be convinced that the expected benefits of the new treatment outweigh the potential risks. The need for further understanding of efficacy and safety should be addressed by specifying the appropriate research questions, and tailoring the study design and analyses to those particular questions. This process must be informed by the challenges arising from the specific clinical context of the new therapy, current available evidence, and patients’ need for timely access to new treatment options. This article has attempted to highlight some general sources of bias related to study design. There may be a risk that access to large amounts of clinical data and sophisticated analytical tools is used to generate results without paying sufficient attention to fundamental methodological concepts that limit what conclusions can be drawn from a specific clinical trial. Attention to methodological basics can help generate reliable study results, and is more important than labelling studies as ‘pragmatic’ or ‘real-world evidence’.

## Disclosure statement

No potential conflict of interest was reported by the authors.
